# *Urtica dioica* Whole Vegetable as a Functional Food Targeting Fat Accumulation and Insulin Resistance-a Preliminary Study in a Mouse Pre-Diabetic Model

**DOI:** 10.3390/nu12041059

**Published:** 2020-04-10

**Authors:** Si Fan, Samnhita Raychaudhuri, Olivia Kraus, Md Shahinozzaman, Leila Lofti, Diana N. Obanda

**Affiliations:** 1 Department of Nutrition and Food Sciences, University of Maryland, College Park, MD 20742, USA; 2 Mathematical and Natural Sciences, College of Computer, University of Maryland, College Park, MD 20742, USA

**Keywords:** *Urtica dioica*, vegetable, diet, fat accumulation, insulin resistance

## Abstract

The shoot of *Urtica dioica* is used in several cultures as a vegetable or herb. However, not much has been studied about the potential of this plant when consumed as a whole food/vegetable rather than an extract for dietary supplements. In a 12-week dietary intervention study, we tested the effect of *U. dioica* vegetable on high fat diet induced obesity and insulin resistance in C57BL/6J mice. Mice were fed ad libitum with isocaloric diets containing 10% fat or 45% fat with or without *U. dioica*. The diet supplemented with *U. dioica* attenuated high fat diet induced weight gain (*p* < 0.005; *n* = 9), fat accumulation in adipose tissue (*p* < 0.005; *n* = 9), and whole-body insulin resistance (HOMA-IR index) (*p* < 0.001; *n* = 9). Analysis of gene expression in skeletal muscle showed no effect on the constituents of the insulin signaling pathway (AKT, IRS proteins, PI3K, GLUT4, and insulin receptor). Notable genes that impact lipid or glucose metabolism and whose expression was changed by *U. dioica* include fasting induced adipocyte factor (FIAF) in adipose and skeletal muscle, peroxisome proliferator-activated receptor-α (Ppar-α) and forkhead box protein (FOXO1) in muscle and liver, and Carnitine palmitoyltransferase I (Cpt1) in liver (*p* < 0.01). We conclude that *U. dioica* vegetable protects against diet induced obesity through mechanisms involving lipid accumulation and glucose metabolism in skeletal muscle, liver, and adipose tissue.

## 1. Introduction

Though they are considered weeds and are rarely a cultivated crop, nettles grow in a broad range of climate conditions and are widespread throughout Europe and North America, North Africa, and in parts of Asia. Nettles (genus *Urtica*) have been used for centuries in traditional medicine in several cultures and have been widely studied as intervention for several medical conditions. Dietary supplements based on *Urtica* spp extracts are widely available. About 25 different species exist within genus Urtica. *Urtica dioica* is the species commonly found in North America, New Zealand, Turkey, and Europe [1]. Actively growing *U. dioica* shoots are harvested before flowering for consumption as an herb or spinach alternative [2,3].

Most studies on *U. dioica* have been performed using its ethanolic or water extract particularly the extract of the leaves and stem which is rich in polyphenols. This extract has been shown to be richer in individual polyphenols than other plants such as dandelion and cranberry juice [1,3].

*U. dioica* extract has been studied for several different disease conditions including autoimmune diseases, such as rheumatoid arthritis, allergy, eczema, atherosclerosis, cancer, as well as age-related degenerative brain disorders. It has also been studied for alleviating bladder disorders, hemorrhaging and inflammation [4,5,6]. More recent studies show that *U. dioica* extract possess anti-diabetic properties [5,6,7]. A comprehensive review on studies using the extract as intervention for diabetes shows that over 21 studies [7]. Obesity induced insulin resistance caused by accumulation of certain lipid metabolites is a key pathophysiologic feature of type 2 diabetes mellitus (T2DM) [8,9]. Progression of insulin resistance into overt type 2 diabetes can be prevented or delayed by timely intervention. Treatment of insulin resistance is directed at increasing insulin sensitivity in peripheral tissues such as skeletal muscle and adipose tissue. Because of the high societal and economic cost of treating diabetes, timely intervention for insulin resistance has important medical, economic, social, and human implications. Therefore, conventional medications that address insulin resistance are a major focus for drug development [10].

Our previous studies using cell culture models showed that *U. dioica* extract activates the proteins AKT1 and AKT2 which are components of the insulin signaling pathway by enhancing their phosphorylation in presence of excess fat metabolites [8,9]. Rather than use the extract, in this study we incorporated whole *U. dioica* vegetable in the diet. Our laboratory focus is on whole foods or class of foods that promote health rather than purified supplements. We thus designed a diet to focus on whole-vegetable effects and formulated the control diets to be isocaloric to the diet containing the vegetable. Furthermore, we tested the efficacy of the vegetable in (i) preventing fat accumulation and insulin resistance when used from the beginning of the study or (ii) reversing fat accumulation and insulin resistance by including it in the diet after obesity is induced with the high fat (HF) diet. We report on effects of the vegetable on a HF diet induced fat accumulation, insulin resistance, inflammation, endotoxemia and the expression of genes encoding proteins that are involved or impact the insulin signaling pathway in the skeletal muscle of C57BL/6J mice.

## 2. Materials and Methods

### 2.1. Acquisition of Plant Sample

*U. dioica* L. (UT) from spring growth was harvested as the total herb above the root mass. The plant was collected from the Green Farmacy Garden a medicinal garden located in Fulton Maryland; latitude 39.1454180, longitude −76.9213600. A voucher sample was deposited at the University Of Maryland herbarium. The stem and leaves were washed, chopped, oven dried (50 °C) for one day, and powdered using a coffee grinder. The powdered sample was subjected to nutrition analysis before being incorporated into the diets.

### 2.2. Nutrition Analysis

The dried powdered sample was analyzed by Medallion Labs (Minneapolis, MN) using the Association of Analytical Chemists (AOAC) methods for calorific value and proximate analysis: moisture, ash, total lipid, protein, carbohydrate, soluble fiber, insoluble fiber, and fatty acid profile. A list of methods used are shown in Supplementary Table S1.

### 2.3. Formulation of Study Diets

We formulated three study diets, the low fat (LF) control, HF control and the HF containing 9% *U. dioica* (HFUT). All diets were formulated to be isocaloric (3961 kcal/Kg) as shown in Table 1. Diets were formulated to be have the same amount of sucrose and protein considering that the *U. dioica* used in this study had 16.1% protein. The LF diet contained 10% fat while the HF and HFUT contained 45% fat.

### 2.4. Study Animals

All animal experiments and procedures were performed in accordance to a protocol approved by the Institutional Animal Care and Use Committee (IACUC) of the University of Maryland. Thirty six (36) male C57BL/6J mice at 7 weeks age were ordered from Jackson Laboratories, Inc. (Bar Harbor, Maine, USA). All mice were singly housed in shoebox cages with corncob bedding in controlled environmental conditions (22 °C), 12 h light dark cycle with *ad libitum* access to food and water.

### 2.5. Randomization and Feeding Regime

After one week baseline feeding of all mice on the LF diet, mice were randomized based on body weight and the homeostatic model assessment of insulin resistance (HOMA-IR) into four groups of 9. Group 1 was fed the LF diet for 12 weeks, group 2 was fed the HF diet for 12 weeks, group 3 was fed the HF diet containing *Urtica dioica* vegetable (HFUT) diet for 12 weeks and, group 4 was fed the HF diet for 6 weeks and then switched to the HFUT diet for the next 6 weeks. Food intake (the difference of weight administered and leftover plus spillage) and body weight were monitored and recorded twice weekly. Sample size of *n* = 9 for each treatment group was used based on a power analysis in our previous animal study on obesity induced insulin resistance [9] which showed 8 to be a number sufficient to generate statistically significant results. To attain a statistical power of 0.90, 8 samples are needed for a one tailed test and 10 samples for a two tailed test.

### 2.6. Determination of Insulin Sensitivity

Fasting (6 h) plasma glucose and insulin were determined at baseline and after 6 and 12 weeks of feeding. Blood was collected by mandibular bleeding. Insulin levels were determined by a mouse ELISA kit (Crystal Chem, Downers Grove, IL). Blood glucose was measured using a portable glucometer (Milipitas, CA, USA). Insulin resistance was assessed by calculating HOMA-IR using the formula: HOMA-IR = fasting glucose (mg/dL) × fasting insulin (ng/mL)/405 as shown before [11].

### 2.7. Tissue Collection and Processing

At 20 weeks of age, following 12 weeks of dietary intervention, animals were anesthetized by isoflurane inhalation. Terminal blood was collected by heart puncture followed by euthanasia by cervical dislocation. Serum was separated and stored at −80 °C to be used in later tests. Abdominal fat pads (epididymal, perirenal, and retroperitoneal) were excised and their weight was determined and summed as total abdominal fat. All tissues collected were snap frozen in liquid nitrogen and then stored at −80 °C for later analysis.

### 2.8. Histopathology

For histopathology, tissue samples were collected and fixed in 10% buffered formalin or snap frozen in liquid nitrogen. The samples fixed in formalin were dehydrated in alcohol and embedded in paraffin before sectioning at a thickness of 2–3 μm. Sections were stained with Oil red O (ORO). The liver snap frozen samples were cut directly in 3–4 μm sections and stained with ORO without any prior fixation (alcohol).

### 2.9. Analysis of Triglycerides in Liver and Colon Contents

About 100 mg of colon contents or 150 mg of liver were separately homogenized in ND40 reagent containing protease inhibitors and analyzed by a triglyceride kit (Cayman Chemical, Ann Arbor MI, USA). Briefly, after centrifuging at 4 °C, the supernatant was diluted 3-fold and reacted with lipoprotein lipase to release glycerol and fatty acids. The glycerol was then quantified by a colorimetric enzymatic reaction according the kit manufacturer’s instructions.

### 2.10. Serum Analysis

Serum samples were analyzed for total triglycerides using a colorimetric kit (Cayman Chemical, Ann Arbor, MI) according to manufactures specifications. Serum LDL cholesterol and HDL cholesterol were quantified using mouse ELISA kits. Endotoxin levels were determined by quantifying serum LPS levels using a mouse ELISA kit. All ELISA kits were from (Cusabio, Wuhan China).

### 2.11. RNA Extraction and cDNA Synthesis

RNA from 50 mg of gastrocnemius skeletal muscle, and liver and 100 mg adipose (epididymal fat pad) was separately extracted and purified using the RNAeasy mini kit (Qiagen, German Town, MD, USA) according to the manufacturer’s specifications. Prior to extraction the samples were disrupted in TRIzol with bead beating using the FastPrep®-24 (MP Biomedical, Solon, Ohio, USA). Both RNA quantity and quality were determined using the Qubit 4 fluorometer (Thermofisher Scientific, RockVille, MD, USA).

Only RNA samples with a concentration over 2.3 ng/uL and an RNA integrity number greater than 7.8 were used for cDNA synthesis using the RT^2^ first strand kit (Qiagen, German Town, MD, USA) with 1000 ng as starting RNA per sample. Similarly, only an RNA with an integrity number greater than 7.8 was used in cDNA synthesis for qPCR of targeted genes.

### 2.12. Analysis of Target Genes in Skeletal Muscle, Adipose Tissue, and Liver by qPCR

Primer sets (IDT Technologies, Coralville IA, USA) were used for analyses of selected genes. The genes selected for this analysis were not exhaustive but we selected genes that have been shown to impact adipogenesis, lipogenesis, fatty acid oxidation, and gluconeogenesis. RT-PCR cycling conditions on the CFX 96 (Bio-rad, Hercules CA, USA), were 2 min at 50 °C and 2 min at 95 °C, followed by 40 cycles of two-step PCR denaturation at 95 °C for 15 s and annealing extension at 60 °C for 1 min. Duplicate assay samples contained 10 ng cDNA and 6 μmol/L primers in 2× PowerUp™ SYBR™ Green Master Mix (Thermofisher Scientific, RockVille, MD, USA) in a final volume of 20 μL. Means of duplicates were taken, and relative amount of target mRNA was normalized to β-actin levels as an endogenous control gene. Data were analyzed according to the 2^−ΔΔCT^ method, and fold difference was calculated between LF, HF, and HFUT groups. A list of primers used is shown in Supplementary Table S1.

### 2.13. Analysis of Insulin Signaling Pathway Genes, by RT^2^ Profiler PCR Array

The differential expression of 84 genes that are involved in, respond to or impact the insulin signaling pathway was determined using the configured RT^2^ Profiler TM PCR Array (Qiagen, German Town, MD, USA), focused on the insulin signaling pathway. About 1000 ng of cDNA from each sample was mixed with SYBR green qPCR master mix and the mixture was then aliquoted in wells of the RT^2^ profiler PCR array. The array was centrifuged for 1 min at 1000× *g* at room temperature before qPCR. Cycling conditions on the CFX 96 (Bio-rad, Hercules CA, USA), were one cycle of 95 °C for 1 min, 40 cycles of 15 s at 95 °C and 60 °C for 60 s. A melting curve analysis was used to verify PCR specificity. The threshold cycle (Cq) values for each gene and that of glyceraldehyde-3-phosphate dehydrogenase (GAPDH) as reference gene were used to calculate ΔΔCq values and relative gene expression was determined by calculating 2^-ΔΔ^*^CT^*. For genes that were downregulated, a reciprocal of 2^−ΔΔ^*^CT^* was calculated. Results were also analysed by the GeneGlobe data analysis center (Qiagen, German Town, MD, USA).

### 2.14. Statistical Analyses

Data are expressed or graphed as mean ± SEM. Differences between two groups were assessed using the unpaired two tailed student *t*-test. Data sets with more than two groups were assessed using the MIXED procedure of SAS 9.4 for analysis of variance between treatments. Main effects were considered significant at *p* ≤ 0.05. Data was analyzed for outliers using studentized residuals. Significant differences observed were followed up using the Bonferroni test of multiple comparisons.

## 3. Results

### 3.1. Nutrition Analysis of U. dioica Dried Powdered Sample

A comprehensive proximate analysis showed that the oven dried powdered leaves and stem sample used for this study contained 5% moisture, 16.1% proteins, 64.1% carbohydrates, 2.2% total fat, and 12.8% ash. Among the carbohydrates 48.3% was insoluble fiber while 4.9% was soluble fiber. Details are shown in Supplementary Table S2. Among the fatty acids in the sample, linoleic, linolenic and palmitic acid constituted 41.8%, 23.1%, and 18.1% respectively. Details are shown in Supplementary Table S2.

### 3.2. Body Weight

**Figure 1:**
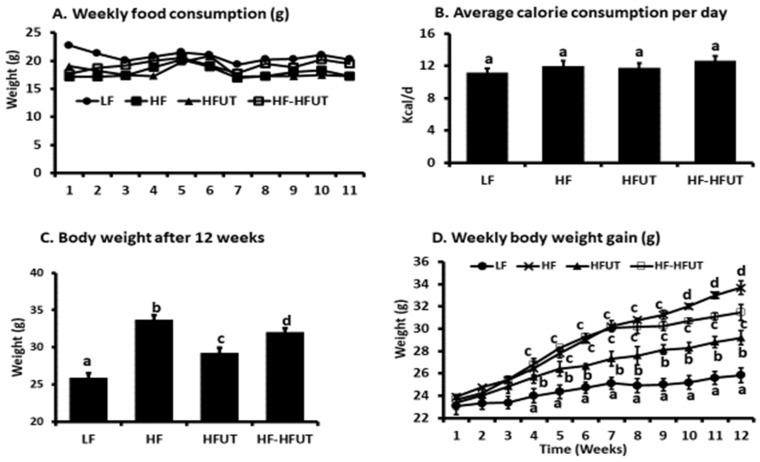
Supplementation with *U. dioica* vegetable lowers body weight. Mice were fed isocaloric diets for 12 weeks. (**A**) Weekly food consumption was not different among all groups. (**B**) The calculated calorie consumption per day was not different among all groups (*p* > 0.05; *n* = 8–9). (**C**) The HF diet induced greater body weight gain compared to LF diet. Supplementation of the HF diet with UT vegetable from beginning of the study lowered body weight compared to the HF diet (*p* < 0.005; *n* = 9). Supplementing the HF diet with UT vegetable after 6 weeks on HF also decreased weight gain by the end of the study compared to HF group (*p* < 0.05; *n* = 8–9). (**D**) Weekly body weight gain was highest in the HF group. The reduction in weight gain was significant by week 4 when UT was included in the HF diet from the beginning of the study. It was significant by week 10 when UT was included in the diet after the first 6 weeks on HF only. ^a, b, c , d^ Different letters indicate a significant difference among treatments at *p* < 0.05.

As expected, the HF diet induced higher weight gain compared to the LF group (*p* < 0.005). The HF diet supplemented with *U. dioica* vegetable (HFUT) had significantly reduced weight gain compared to the HF diet over the 12 weeks (Figure 1C). Switching the diet from HF to HFUT after 6 weeks in group 4 also significantly reduced weight gain to levels lower than those of the HF diet (*p* < 0.05) (Figure 1C). While weight gain was similar in groups 2 and 4 in the 1st 6 weeks of the study, switching group 4 to HFUT significantly lowered weekly weight gain during the last 6 weeks (*p* < 0.05) (Figure 1C,D). The significant reduction in weight gain in group 4 was evident in week 10 (4 weeks after switching to HFUT) (Figure 1D).

### 3.3. Food Consumption and Energy Intake

Weekly food consumption and the calculated average energy intake per day were not significantly different between the four treatment groups (*p* > 0.05; *n* = 9) (Figure 1A,B).

### 3.4. Fasting Glucose, Fasting Insulin, and Insulin Resistance

**Figure 2:**
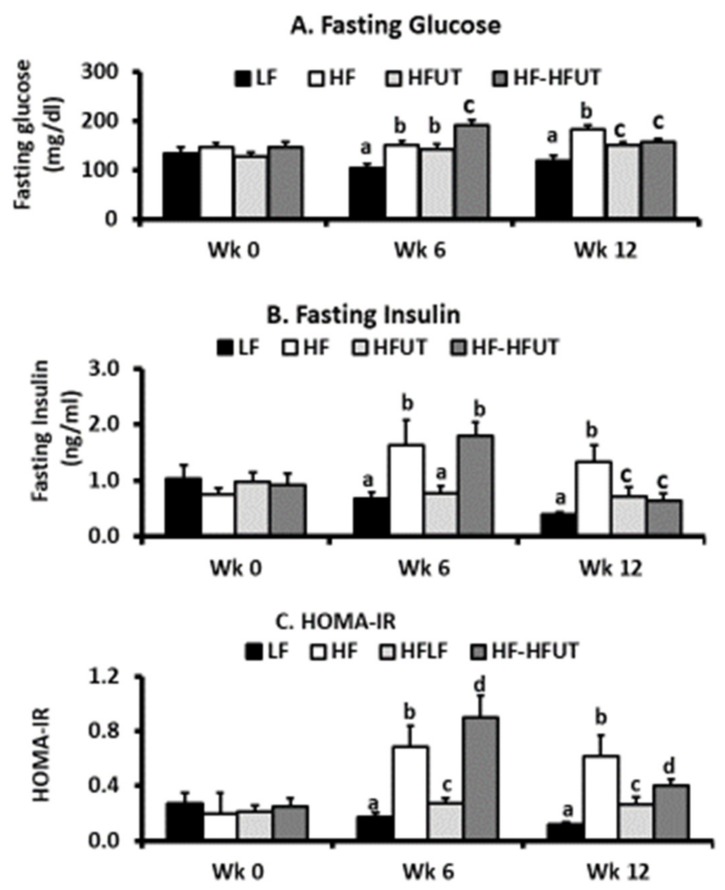
Supplementation with *U. dioica* vegetable reduced fasting glucose, fasting insulin, and enhanced insulin sensitivity. Fasting glucose and fasting insulin were determined by glucometer and ELISA test respectively. (**A**) At week 0 (baseline), all groups had similar fasting glucose. By week 6 the HF diet induced higher fasting glucose and UT had no effect. By week 12, the HF fed group had the highest fasting glucose and UT vegetable induced significantly lower glucose levels in group 3 (fed UT for 12 weeks) and group 4 (fed UT for 6 weeks) compared to HF group (*p* < 0.05; n = 8–9). (**B**) At baseline, all groups had similar fasting Insulin. By week 6 the HF diet induced higher fasting glucose in the two groups and group 3 fed with UT had significantly lower insulin levels compared to the HF group. By week 12, the HF fed group had the highest fasting glucose and UT vegetable induced significantly lower insulin levels in group 3 (fed UT for 12 weeks) and group 4 (fed UT for 6 weeks) compared to HF group (*p* < 0.05; *n* = 8–9). (**C**) At baseline, all groups had similar HOMA-IR values. By week 6, the HF diet induced higher HOMA-IR and group 3 fed with UT had significantly lower HOMA-IR (*p* < 0.001; *n* = 8–9). By week 12, the HF fed group had the highest HOMA-IR and UT vegetable induced significantly lower insulin levels in group 3 (fed UT for 12 weeks) and group 4 (fed UT for 6 weeks) compared to HF group (*p* < 0.05; *n* = 8–9). ^a, b, c, d^ Different letters indicate a significant difference among treatments at *p* < 0.05.

As expected, the HF diet induced significantly higher fasting glucose compared to that of the LF group within 6 weeks. At the 6-week time frame fasting glucose in the HFUT diet was at the same level as that of the HF group (*p* > 0.05). However, at the 12-week time frame, the groups fed HFUT diet both had lower fasting glucose than the HF only group (*p* < 0.05) (Figure 2A).

At the 6-week time point, the mice fed the HF diet (groups 2 and 4) both had significantly higher fasting insulin compared to that of the LF group (*p* < 0.05). At this time frame, fasting insulin in group 3 fed HFUT diet was the same as that of the LF group (*p* > 0.05). At the 12-week time frame, the groups fed HFUT diet both had lower fasting insulin compared to the HF only group (*p* < 0.05) (Figure 2B). Feeding the mice HFUT over 12 weeks or introducing HFUT only in the last 6 weeks after inducing obesity with HF had the same effect on fasting insulin (Figure 2B).

At the 6-week time point, the mice fed the HF diet (groups 2 and 4) both had significantly higher HOMA-IR compared to the LF group (*p* < 0.05). At this time, group 3 fed the high fat supplemented with the vegetable (HFUT) diet had the same fasting HOMA-IR as the LF group (*p* > 0.05). At the 12-week time frame, the groups fed HFUT diet both had lower HOMA-IR compared to the HF only group (*p* < 0.05) (Figure 2C). Feeding HFUT over 12 weeks or introducing HFUT in the last 6 weeks after inducing obesity with the HF diet lowered HOMA-IR by the end of the study (Figure 2C).

### 3.5. Fat Accumulation in Adipose Tissue

**Figure 3:**
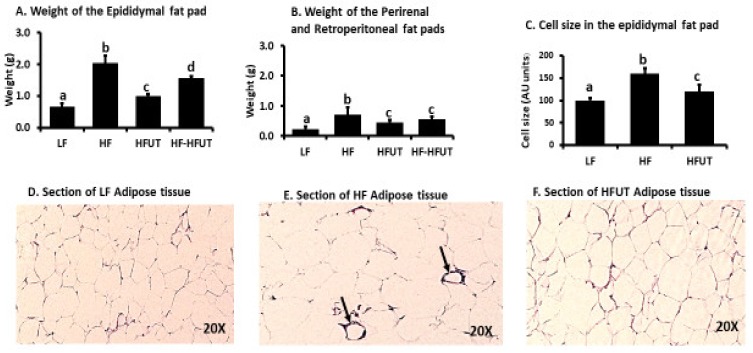
Supplementation with *U. dioica* L. (UT) vegetable lowers fat accumulation and inflammatory markers in adipose tissue. (**A**–**B**) The weight of the epididymal fat pad and combined weight of perirenal and retroperitoneal fat pads were higher in HF compared to LF fed group (*p* < 0.05; *n* = 9). Supplementation of the HF diet with UT vegetable from beginning of the study, lowered overall fat pad weights compared to the HF diet (*p* < 0.05; *n* = 9). Supplementing the HF diet with UT vegetable after 6 weeks on HF also decreased fat pad weight by the end of the study compared to HF group (*p* < 0.05; *n* = 8–9). (**C**) Cell size of epididymal fat H and E stained sections was determined by measuring cross-sectional width of the cells at 40X magnification and standardizing LF group size to 100. (**D**–**F**) H and E stained sections of the epididymal fat pad showed that LF group had smaller adipocyte in size with no markers of inflammation visible. The HF sections showed larger adipocytes and presence of crown like structures the markers of inflammation (shown by arrow). The HFUT sections showed smaller adipocytes and no crown like structures. ^a, b, c^ Different letters indicate a significant difference among treatments at *p* < 0.05.

The sum of the fat pads (epidydimal, retroperitoneal and perirenal) for each mouse showed that the HF diet induced fat accumulation significantly higher than that in the LF group (*p* < 0.05; *n* = 9). The HFUT (group 3) had significantly reduced fat weight compared to the HF (group 2) over the 12 weeks (Figure 3A,B). Switching the diet from HF to HFUT in group 4 also significantly reduced fat pad weight to levels lower than those of the HF diet only (*p* < 0.05).

The HF diet induced an increase in adipocyte size and appearance of crown like structures (histologic hallmarks of the proinflammatory process) in epididymal fat. Adipose tissue of HFUT had smaller size adipocytes and sparse crown like structures and looked similar to that of LF diet (Figure 3C–E).

### 3.6. Liver Weight and Lipids

**Figure 4:**
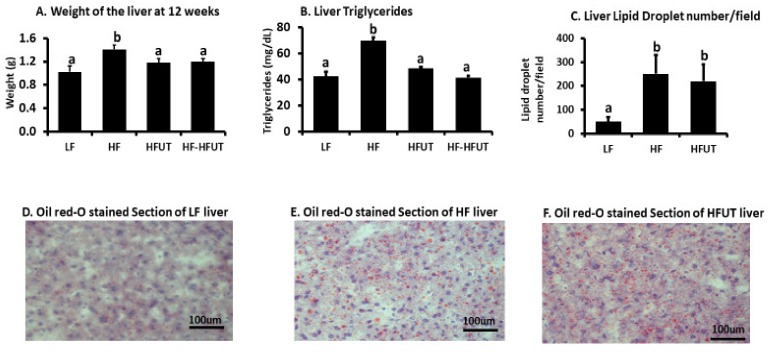
Supplementation with UT vegetable lowers liver weight and liver triglycerides but not liver lipid droplet number. (**A**) The weight of the liver was higher in HF compared to LF fed group (*p* < 0.05; *n* = 9). Supplementation of the HF diet with UT vegetable from beginning of the study or after 6 weeks of HF feeding lowered liver weight to levels similar to that of the low fat (LF) (*p* < 0.05; *n* = 8–9). (**B**) Liver triglycerides were higher in HF compared to LF fed group (*p* < 0.05; *n* = 9). Supplementation of the HF diet with UT vegetable from beginning of the study or after 6 weeks of HF feeding lowered liver triglycerides to levels similar to those of the LF group (*p* < 0.05; *n* = 8–9). (**C**–**F**) Oil red O (ORO) stained liver slides from HF fed mice showed highly increased lipid droplets compared to the LF group. The lipid droplet number in the HFUT group was not different from that of the HF group. ^a,b^ Different letters indicate a significant difference among treatments at *p* < 0.05.

The HF diet induced increased liver weight, and the HFUT diet reduced liver weight in both groups 3 and 4 to levels significantly lower than those of the HF group but not different from the LF group (*p* < 0.05) (Figure 4A). The diet with UT reduced liver triglycerides in both groups 3 and 4 to levels different from the HF group (*p* < 0.005) but not different from the LF (Figure 4B). The ORO stained sections of the liver showed more lipid droplets in liver of HF group compared to LF. Liver from HFUT mice had similar amounts of lipid droplets like those of HF (Figure 4C–F).

### 3.7. Lipid Analysis in Serum and Colon Contents

The HF diet increased in triglycerides in serum and colon contents compared to the LF (*p* < 0.05). The levels of triglycerides in serum and colon contents in both HFUT fed groups trended lower but were not significantly different from those of the HF diet fed group (*p* > 0.05) (Table 2). The LF diet had higher levels of LDL cholesterol compared to HF group (*p* < 0.05). The HFUT group also had no effect on LDL cholesterol levels; not significantly different from the HF group (*p* > 0.05). Serum of HF mice had significantly lower HDL cholesterol than LF group and the HDL cholesterol levels in HF and HFUT groups were not different (Table 2).

### 3.8. Endotoxemia Markers in Serum

The high fat diet increased LPS concentrations in serum. The HFUT diet had no effect on serum LPS; results were not different from those of HF diet (*p* > 0.05) (Table 2).

### 3.9. Gene Expression by qPCR Array

**Figure 5:**
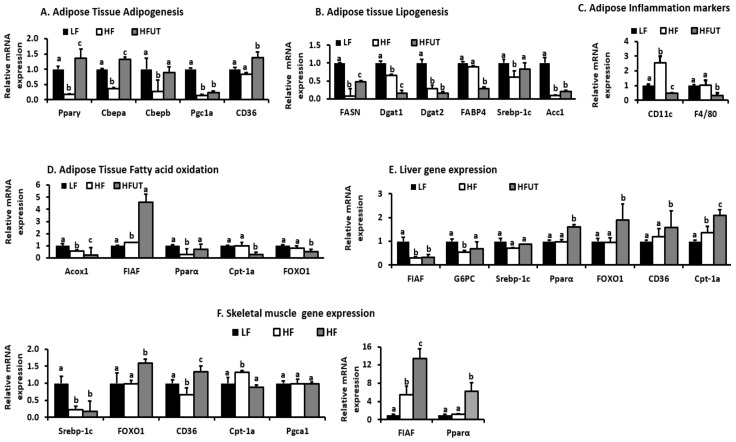
Changes in mRNA expression of genes that are involved or impact insulin signaling in skeletal muscle. RNA was extracted, cDNA synthesized, and gene expression determined by qPCR. The threshold cycle (Cq) values for each gene and that of β-actin as reference gene were used to calculate gene expression by calculating 2^-ΔΔ^*^CT^*. (**A**) Genes involved in adipose tissue adipogenesis. (**B**) Genes involved in adipose tissue lipogenesis. (**C**) Markers of inflammation in adipose tissue. (**D**) Genes involved in fatty acid oxidation in adipose tissue (**E**) Gene expression in Liver. (**F**) Gene expression in Skeletal muscle. ^a, b, c^ Different letter indicate a significant difference among treatments (*p* < 0.05; *n* = 3).

In Figure 5, we show the mRNA expression of selected genes in the HF and HFUT groups relative to the LF control. In adipose tissue the HF diet lowered the expression of genes involved in adipogenesis; peroxisome proliferator-activated receptor gamma (Pparγ), CCAAT/enhancer binding protein alpha (C/EBPa), C/EBPb, peroxisome proliferator-activated receptor gamma coactivator 1-alpha (Pgc1a) (*p* < 0.05; *n* = 3). The HFUT diet reversed this reduction and also enhanced the expression of CD36 (Figure 5A). Compared to the HF diet, HFUT lowered the expression of fatty acid synthase (Fasn), diacylglycerol O-acyltransferase 1 (Dgat1), Dgat2, Acetyl-CoA carboxylase (ACC1), and fatty acid binding protein 4 (FABP4) all markers of lipogenesis and triglyceride synthesis (*p* < 0.05; *n* = 3) (Figure 5B). The HF diet increased the expression of CD11c by over 2-fold but had no effect on F4/80 and the HFUT lowered these two genes to levels below those of the LF diet (*p* < 0.05; *n* = 9). The HFUT diet caused a 6-fold increase in fasting induced adipocyte factor (FIAF) (*p* < 0.001; *n* = 3) and attenuated the HF diet induced reduction in the gene expression of Pparα (*p* < 0.05; *n* = 3) (Figure 5D). HFUT diet lowered expression of Peroxisomal acyl-coenzyme A oxidase 1 (Acox1), carnitine acyltransferase I (Cpt-1a) and Forkhead box protein O1 (FOXO1) (*p* < 0.05).

In the liver, HFUT increased expression of Ppar-α, FOXO1, Cpt-1a all considered markers of fatty acid oxidation and increased the expression of CD36 (*p* < 0.05). HFUT had no effect on FIAF, Glucose-6-phosphatase, catalytic subunit (G6PC) and Sterol regulatory element-binding transcription factor 1c (Srebpc-1c) in liver. In skeletal muscle, HFUT had no effect on Pgc1a and Srebpc-1c but increased FOXO1, CD36, Cpt-1a, FIAF and Pparα. FIAF was increased by 13-fold while Pparα was increased by 7 fold (*p* < 0.001; *n* = 3).

### 3.10. Gene Expression of Insulin Signaling Genes in Skeletal Muscle by qPCR Array

In Supplementary Table S4, we show the relative mRNA expression values of genes that are involved in or impact the insulin signaling pathway in skeletal muscle. We show values of the LF group (as control) relative to the HF group, HF group (as control) relative to the HFUT, and the LF group (as control) group relative to the HFUT group.

## 4. Discussion

Although diet supplements based on *Urtica dioica* extracts are widely available in the United States, not much is known about the benefits of this plant when consumed as a whole vegetable. In many international cultures *U. dioica* shoots are harvested before flowering for use as an herb or spinach alternative. Some recipes incorporate *U. dioica* leaf flour in bread, pasta, and noodle dough. Besides being high in polyphenols, on a dry weight basis, *U. dioica* leaf is comparable to common bean (*Phaseolus vulgaris*) and chicken as a source of essential amino acids [1,2,3]. In the current study, we show that including the vegetable in a HF diet, attenuates fat accumulation in adipose tissue and whole-body insulin resistance. In our previous study [8] in which we incorporated 0.5% *U. dioica* ethanolic extract into the HF diet, we did not observe a reduction in body weight or body composition; the extract improved insulin signaling despite not having an effect on fat accumulation. Our current findings on reduction of body weight and fat accumulation when the whole vegetable is used instead of the extract, are thus novel and raise new questions on mechanisms involved. It also suggests that other components of the vegetable besides the phytochemicals in the ethanol extract may impact positive effects on body weight.

The benefits of the *U. dioica* vegetable are likely attributed to the high fiber content of 53.3% (Supplementary Table S2), phytochemical components, high protein content, unique fatty acid profile and high percentage of linoleic and linolenic acids (Supplementary Table S3). The soluble and insoluble fiber may contribute to beneficial effects through the action of the gut microbiota. The wide range of compounds in the ethanolic extract have been elucidated by a number of researchers [1,12,13] and have been shown to include the phenolic acids; p-hydroxybenzoic acid, gentisic acid, protocatechuic acid, vanillic acid, quinic acid, ferulic acid, p-coumaric acid, caffeic acid and, 5-*O*-caffeolylquinic acid. The second large group of compounds are flavonols which include kaempferol, kaempferol 3-*O*-glucoside, quercitrin, quercetin 3-*O*-glucoside, quercetin 3-*O*-rutinoside (rutin), and isorhamnetin. Others are the biflavonoids amentoflavone and the catechin a flavan-3-ol. All these phytochemicals and the fiber content may individually impact obesity and insulin resistance in ways that are yet unknown.

The connections between obesity and metabolic diseases are well established. Obesity leads to elevated levels of circulating free fatty acids that are converted into metabolites that induce insulin resistance [8,9] and leads to low-grade elevation in plasma of gut derived lipolysaccharide (LPS) (metabolic endotoxemia) [14,15]. When bound to its receptor Toll-Like Receptor 4, LPS stimulates whole-body and tissue specific metabolic perturbations by initiating a signaling cascade that results in pro-and anti-inflammatory pathways and initiates obesity and insulin resistance. The HF diet significantly raised LPS levels in blood but supplementation with *U. dioica* vegetable did not lower LPS levels (Table 2). It is likely that the vegetable attenuates fat accumulation and insulin resistance by mechanisms not related to bacterial LPS.

In agreement with our previous study using the water/ethanol extract of *U. dioica* [8], UT vegetable prevented the development of insulin resistance in mice fed the HF diet and also reversed insulin resistance when obese mice were switched to the vegetable containing diet (Figure 2). The HFUT diet significantly attenuated fat accumulation in adipose tissue and resulted in smaller adipocytes with no evidence of inflammatory markers (crown like structures) (Figure 3). This is in line with previous work which shows that when adipocytes enlarge (hypertrophy) in obesity, they release TNFα and insulin-like growth factor which stimulate hyperplasia. Reversal of obesity decreases the number and size of the adipocytes followed by apoptosis of adipocytes [16]. The positive results of fat accumulation in liver were inconclusive; while liver weight and liver triglycerides were lowered by the *U. dioica* (Figure 4), the effect on serum triglycerides and cholesterol levels was not different from those of the HF diet (Table 2). However, the mice were not fasted before euthanasia. The results may have been different if terminal blood and tissues were collected after fasting. An unexpected observation was the higher amount of LDL cholesterol in the LF diet compared to the HF diet. The LF diet had a higher amount of carbohydrates compared to the HF diet. It has been previously shown that increased dietary carbohydrates, particularly simple sugars and starches with high glycemic index, can increase levels of small, dense LDL and HDL [17]. The increased triglycerides observed in colon contents of HF diet fed mice was not lowered by the diet with *U. dioica* vegetable (Table 2). It is likely that while *U. dioica* impacts fat metabolism and assimilation in ways that are yet unknown, it does not affect fat digestion or absorption in the digestive tract.

We evaluated the effect of HFUT on the expression of genes that encode proteins involved in inflammation, fatty acid transport, fatty acid oxidation, adipogenesis, and lipogenesis. The HF diet induced a more than 2-fold increase in CD11c compared to the LF diet group. The HFUT reversed this and down regulated CD11c to levels below those of the LF diet group. HFUT also reduced expression of F4/80 another marker of inflammation in adipose (Figure 5C). A specific subset of macrophages that expresses CD11c and produces high levels of pro-inflammatory cytokines is recruited to obese adipose and muscle tissue. These macrophages are linked to the development of obesity-associated insulin resistance. Using a conditional cell ablation system, to deplete CD11c^+^ cells in obese mouse models Patsouris et al. [18] showed that depleting CD11c^+^ cells results in rapid normalization of insulin sensitivity and leads to a marked decrease gene expression and protein levels of inflammatory markers. CD11c plays an important role in T-cell accumulation and activation in adipose tissue contributes to insulin resistance associated with obesity. CD11c messenger RNA positively correlates with MCP-1 in visceral adipose of obese humans with metabolic syndrome compared with lean humans [19]. Thus CD11c^+^ cells are a potential therapeutic target for treatment of obesity-related insulin resistance and type II diabetes.

Compared with the HF diet, HFUT increased the expression of genes that are involved in fatty acid transport and oxidation in both adipose tissue and skeletal muscle. Specifically, the expression of Angiopoietin-like protein or fasting induced adipocyte factor (FIAF) increased more than 4 fold in adipose and by 13 fold in muscle. FIAF is a circulating inhibitor of lipoprotein lipase and restrains its ability to import and store fatty acids in peripheral tissues. Furthermore, FIAF expression regulates energy expenditure via modulation of AMP-activated protein kinase (AMPK) activity in muscle and adipose. Elevated FIAF levels have been shown to protect against diet-induced obesity [20]. This may be a contributing factor in the lower fat accumulation observed in HFUT fed mice. The HFUT increased the expression of Ppar-α in skeletal muscle by more than 6-fold and increased it by 1.6 fold in liver. In adipose tissue, the HFUT prevented the HF induced reduction in expression of Ppar-α and increased Ppar-γ expression by 1.4 fold (Figure 5D–F). Activators of both PPARα and PPARγ have been shown to improve insulin sensitivity and normalize impaired glucose tolerance in both humans and rodent models of IR. In liver, PPAR-α regulates genes involved in lipid and lipoprotein metabolism. When activated, it promotes fatty acid oxidation by inducing expression of mitochondrial acyl-CoA dehydrogenases, acetyl-CoA production, promotes glucose sparing and ketone body synthesis by upregulating mitochondrial hydroxymethylglutaryl-CoA synthase (HMGS), a rate-limiting ketogenesis enzyme [21,22]. It also reduces pyruvate kinase, induces PDK4 expression and reduces weight gain in rodents [21,22]. PPARα activation further reduces plasma triglyceride-rich lipoproteins by increasing the activity of LPL, which hydrolyzes lipoprotein triglycerides. Thus, through enhanced fatty acid oxidation, UT vegetable may impact fat accumulation mechanisms and result in the leaner phenotype. The HF diet lowered PPARγ expression and the HFUT prevented this and enhanced the expression to levels higher than those of the LF group. PPARγ is highly expressed in adipose tissue where its activation alters fat topography, adipose phenotype and upregulates genes involved in fatty acid metabolism and triglyceride storage.

In liver and muscle, the HFUT diet increased FOXO1 gene by almost 2 fold (Figure 5E,F). FOXO proteins are important in metabolism and energy homeostasis. FOXO1 is particularly inhibits hepatic gluconeogenesis by insulin action. Insulin inhibits FOXO1 activity through the PI3K/ AKT signaling pathway resulting in an inhibition of gluconeogenesis by suppressing expression of glucose-6-phosphatase (G6PC) and Pck1 [23]. FOXO1 stimulates fatty acid uptake and oxidation in muscle cells. Other genes involved in fatty acid oxidation and energy expenditure and impacted by HFUT were fatty acid translocase (FAT/*CD36*), carnitine palmitoyl transferase (CPT-1a) (by more than 2 fold in liver), FABP and Acc2. Overexpression of Cpt1 in skeletal muscle has been shown to enhance fatty acid oxidation and improve high-fat diet-induced insulin resistance [24]. The expression of CD36 was enhanced by HFUT in all three tissues. CD 36 promotes adipocyte differentiation and adipogenesis.

Overall, the HFUT diet increased the expression of genes involved in adipogenesis (Pparγ, CEBPα, SREBF1c, FABP4, FASN, and CD36), lowered genes involved in lipogenesis (Acc1, Dgat1, Dgat2, FABP) and increased genes involved in fatty acid oxidation (FIAF, Pparα, Cpt-1). Thus the combination of reduction in lipogenesis genes and increase in and fatty acid oxidation genes contributed to the leaner frame in HFUT fed mice.

The analysis of genes that impact the insulin signaling pathway in skeletal muscle using the RT^2^ array showed that HFUT diet did not induce significant changes in the expression in most of the 84 genes. (Supplementary Table S4). In agreement with our previous study [8] we found no significant change in the expression of AKT1, AKT2, and AKT3 isoforms and GLUT4 (Slc27a4). In our previous studies [8,9] we showed that *U. dioica* extract enhanced insulin signaling by enhancing the phosphorylation of AKT1 and AKT2 but had no effect on their gene or protein expression. The notable few changes observed using the RT^2^ array include the reduction in uncoupling protein 1 (mitochondrial proton carrier) (UCP1), increase in eukaryotic translation initiation factor 4E binding protein 1 and increase in glucose-6-phosphatase, catalytic (GPC6). These genes are expressed in very low amounts in skeletal muscle and hence their changed expression may not impact mechanisms. Furthermore, we were unsuccessful in confirming the changed expression of UCP-1 and G6PC using qPCR even after using primers validated by IDT. The results of Srebp1C were replicated in the qPCR assays. The HFUT diet downregulated glucose 6-phosphatase (G6PC), the gene that encodes the enzyme that dephosphorylates the glucose molecule to enable its transport out of the cells. Primarily expressed in the liver, the enzyme is present in muscle cells in very low amounts. The downregulation of this gene by HFUT diet may augment its role in keeping glucose within the muscle cells and thus result in lower blood glucose levels. The HF diet lowered the levels of eukaryotic translation initiation factor 4E binding protein 1 (Eif4ebp1) gene but supplementing the HFUT prevented this. Increased insulin resistance in 4E-BP1 and 4E-BP2 KO mice is linked to increased ribosomal protein S6 kinase (S6K) activity and impairment of AKT signaling in muscle, liver, and adipose tissue [25]. The HFUT diet downregulated UCP1 in skeletal muscle. UCP1 is mainly expressed in brown adipose tissue where its role in thermogenesis has been widely studied. Although it is now known that UCP1 is also expressed in white adipose and skeletal muscle, its physiological functions in these tissues is not well established and different studies had shown conflicting results. Our findings on downregulation of UCP1 in skeletal muscle contrast with a number of studies that have linked overexpression of UCP1 to increased energy expenditure, reduced fat mass and improved glucose tolerance [26,27,28]. However, the overexpression of UCP1 does not reduce susceptibility to obesity when the mice are not challenged thermogenically [26]. In fact, obesity-resistant UCP1-deficient mice have increased resistance to obesity due to the inactivation of a major thermogenic mechanism that results in an increased expenditure of energy [26].

Our data clearly shows a relationship between the effects of *U. dioica* vegetable and favorable metabolic parameters but raises several unanswered questions. The limitation of our study is that we assess mRNA changes (transcript abundance) as a proxy for protein levels but have not yet quantified changes in the proteins encoded by these genes. Protein expression levels are highly regulated and protein levels are more conserved than the mRNA levels. In addition to changes in mRNA expression, differences in the translational efficiency of the mRNAs are important. In further studies we shall evaluate protein expression levels, identify the active plant components responsible for the positive effects and metabolites produced in tissues to gain a better understanding of the potential of *U. dioica* as a metabolically favorable functional whole food for obesity and insulin resistance.

## Figures and Tables

**Table 1:** Diet Formulation.

	LF	HF	HFUT
**Ingredient (g)**			
Casein	200	200	185.3
l-Cystein	3	3	3
Corn starch	452.2	72.8	63.94
Maltodextrin 10	75	100	100
Sucrose	172.8	172.8	172.8
Cellulose	50	50	6.34
Soybean oil	25	25	23.16
Lard	20	177.5	177.5
Mineral Mix	10	10	10
Di calcium phosphate	13	13	13
Calcium carbonate	5.5	5.5	5.5
Potassium citrate	16.5	16.5	16.5
Vitamin mix	10	10	10
Choline Bitartrate	2	2	2
*Urtica dioica* dried powdered shoot	0	0	82
Total weight (g)	1055.05	858.15	870.82
kcal			
Protein	716	716	716
Carbohydrate	2840	1422.4	1422.4
Fat	405	1822.5	1822.5
TOTAL kcal	3961	3960.9	3961

**Table 2:** Serum and colon content analyses.

	LF	HF	HFUT	HF-HFUT
***Triglycerides***					
Colon contents (mg/dL)	31.1 (1.49) ^a^	55.1(4.23) ^b^	51.7 (2.23) ^b^	52.6 (4.61) ^b^	0.021
Serum	187.9 (30.6) ^a^	259.7 (50.8) ^b^	199.6 (43.5) ^b^	176.7 (24.5) ^b^	0.047
***Serum*** ***Cholesterol***					
LDL-Cholesterol (mg/mL)	1.57 (0.16) ^a^	1.07 (0.14) ^b^	1.08 (0.09) ^b^	−	0.046
HDL-Cholesterol (mg/mL)	0.82 (0.026) ^a^	0.77 (0.014) ^a^	0.72 (0.03) ^a^	−	0.09
***LPS***					
Serum LPS (ng/mL)	1.38 (0.63) ^a^	1.66 (0.29) ^b^	1.78 (0.25) ^b^	−	0.04

Each value represents the mean (SE) ^a, b^ Different letters indicate a significant difference among treatments at *p* < 0.05.

## Supplementary Materials

The following are available online at https://www.mdpi.com/2072-6643/12/4/1059/s1, Table S1: Primers used for qPCR, Table S2: Nutrient Analysis of leaves and stems of *Urtica dioica L,* Table S3: Fatty acid profile of the leaves and stems of *Urtica dioica L,* Table S4: Relative fold changes in expression of genes that are involved or impact insulin signaling in skeletal muscle.
