# Supplementary material for: Urtica dioica Whole Vegetable as a Functional Food Targeting Fat Accumulation and Insulin Resistance-a Preliminary Study in a Mouse Pre-Diabetic Model
Source: Nutrients. 2020 Apr 10;12(4):1059. doi: 10.3390/nu12041059 (PMC7231388; doi:10.3390/nu12041059)
Supplement: Supplementary file 1 [file nutrients-12-01059-s001.pdf]

Supplementary Table 1. Primers used for qPCR.

	Gene Symbol	Forward	Reverse Primer
1.	B-Actin	5-GGCACCACACCTTCTACAATG-3′	5′-GGGGTGTTGAAGGTCTCAAAC-3′
2.	FIAF/Angptl4	5′-TTTCCCTGCCCTTCTCTACT-3′	5′-GTACCAAACCACCAGCCA-3′
3.	UCP-1	5′-CAAATCAGCTTTGCCTCACTC-3′	5′-CACACCTCCAGTCATTAAGCC-3′
4.	G6PC	5′-TCTACCTTGCTGCTGCTCACTTTC-3′	5′-GGAGGCTGGCATTGTAGATG-3′
5.	Srebp-1c	5′-GAACAGACACTGGCCGAGAT-3′	5′-GAGGCCAGAGAAGCAGAAGAG-3′
6.	Ptl	5′-CCTGAACAGTGCTGAAGTGAG-3′	5′-ACAGTACACATTTACTGCATCCC-3′
7.	Pgc1a	5′-CTCCATGCCTGACGGCACCC-3′	5′-GCAGGGACGTCTTTGTGGCT-3′
8.	Ppara	5′-ATGCCAGTACTGCCGTTTTCA-3′	5′-GGGCCACAGAGCACCAATCTGTG-3′
9.	PPARg	5′-GTGCCAGTTTCGATCCGTAGA-3′	5′-GGCCAGCATCGTGTAGATGA-3′
10.	CD36	5′-TGGCCTTACTTGGGATTGG-3′	5′-CCAGTGTATATGTAGGCTCATCCA-3′
11.	Foxo1	5′-ACATTTCGTCCTCGAACCAGCTCA-3′	5′-ATTTCAGACAGACTGGGCAGCGTA-3′
12.	F4/80	5′-CTTTGGCTATGGGCTTCCAGTCC-3′	5′-GCAAGGAGGACAGAGTTTATCGTG-3′
13.	FASN	5′-AGCGGCCATTTCCATTGCCC-3′	5′-CCATGCCCAGAGGGTGGTTG-3′
14.	ACC1	5′-ACAGTGGAGCTAGAATTGGAC-3′	5′-ACTTCCCGACCAAGGACTTTG-3′
15.	Cpt-1a	5′-CAGAGGATGGACACTGTAAAGG-3′	5′-CGGCACTTCTTGATCAAGCC-3′
16.	Dgat1	5′-TGGCTGCATTTCAGATTGAG-3′	5′-GCTGGGAAGCAGATGATTGT-3′
17.	Dgat1	5′-CTTCCTGGTGCTAGGAGTGGC-3′	5′-GCTGGATGGGAAAGTAGTCTCGG-3′
18.	CD11c	5′-CTGGATAGCCTTTCTTCTGCTG-3′	5′-GCACACTGTGTCCGAACTCA-3′
19.	Cbep-a	5′-AAACAACGCAACGTGGAGAC-3′	5′-TGTCCAGTTCACGGCTCAG-3′
20.	Cbep-b	5′-GGGTTGTTGATGTTTTTGGTTT-3′	5′-GAAACGGAAAAGGTTCTCAAAA-3′
21.	Acox1	5′-TCGAAGCCAGCGTTACGAG-3′	5′-ATCTCCGTCTGGGCGTAGG-3′

Supplementary Table 2. Nutrient Analysis of leaves and stems of *Urtica dioica*.

	Amount %	Analytical Method used
Proximate Analysis		
Ash	12.8	AOAC: 923.03
Carbohydrates	64.1	By Calculation
Protein (6.25)	16.1	AACC 46-30*; AOAC 992.15
Total Fat	2.2	AOAC: 996.06
Fiber		
Insoluble Dietary Fiber	48.3	AOAC: 991.43*
Soluble Dietary Fiber	4.9	
Total dietary Fiber	53.3	
Fat		AOAC: 996.06*
Saturated Fat	0.54	
Monounsaturated Fat	0.16	
cis-cis polyunsaturated fat	1.40	
Trans fat	0.05	
Moisture	5.0	AOAC: 945.43*, 934.01
Calories per 100g		
Calories	340.26	By calculation
Calories, 2020	136.74	
Calories from Fat	20.67	
Calories from Saturated Fat	4.77	
Calories (insoluble fiber subtracted)	14.31	

Supplementary Table 3: Fatty acid profile of the leaves and stems *Urtica dioica.*

Component Name	Normalized by Weight	% (w/w) as Triglyceride in Product
C-12:0 Lauric	1.52%	0.021
C-16:0 Palmitic	18.0%	0.252
C-16:1 t-Hexadecenoic	1.50%	0.021
C-18:0 Stearic	2.24%	0.031
C-18:1 t-Elaidic	0.75%	0.010
C-18:1 Oleic	7.45%	0.104
C-18:2 Linoleic	41.76%	0.585
C-20:0 Arachidic	0.74%	0.010
C-18:3 Linolenic	23.12%	0.324
C-22:0 Behenic	0.74%	0.010
C-20:4 Arachiodonic	0.74%	0.010
C-23:0 Tricosanoic	1.48%	0.021
**Totals:**	**100%**	**1.40**

Supplementary Table 4. Relative fold changes in expression of genes that are involved or impact Insulin signaling in skeletal muscle.

Insulin signaling	Symbol	LF Vs HF	HF VS HFUT	LF Vs HFUT
Acetyl-Coenzyme A carboxylase alpha	Acaca	-1.07±0.4	-1.36±0.3	-1.28±0.4
Acyl-Coenzyme A oxidase 1, palmitoyl	Acox1	1.01±0.06	-1.31±0.18	-1.21±0.01
Adrenergic receptor, alpha 1d	Adra1d	1.1±0.07	-1.08±0.6	1.01±0.2
AE binding protein 1	Aebp1	-1.36±0.3	1.04±0.09	-1.30±0.4
Thymoma viral proto-oncogene 1	Akt1	1.06±0.04	-1.00±0.01	1.06±0.03
Thymoma viral proto-oncogene 2	Akt2	1.00±0.1	-1.22±0.2	-1.21±04
Thymoma viral proto-oncogene 3	Akt3	1.02±0.04	1.02±0.03	1.04±0.07
V-raf murine sarcoma 3611 viral oncogene homolog	Araf	-1.10±0.3	-1.10±0.07	-1.22±0.5
Bcl2-like 1	Bcl2l1	-1.00±0.3	-1.21±0.3	-1.21±0.4
Braf transforming gene	Braf	-1.22±0.5	-1.17±0.3	-1.43±0.6
CAP, adenylate cyclase-associated protein 1 (yeast)	Cap1	-1.12±0.06	-1.07±0.3	-1.20±0.2
Casitas B-lineage lymphoma	Cbl	-1.87±0.6	-1.04±0.3	-1.95±0.7
CCAAT/enhancer binding protein (C/EBP)α	Cebpa	-1.11±0.2	1.15±0.8	1.45±0.6
CCAAT/enhancer binding protein (C/EBP)β	Cebpb	-1.24±0.2	1.21±0.3	1.03±0.6
Complement factor D (adipsin)	Cfd	-1.21±0.01	-1.41±0.2	-5.95±1.6
Docking protein 1	Dok1	-1.16±0.5	-1.09±0.01	-1.27±0.6
Docking protein 2	Dok2	-1.67±0.1	1.27±0.6	-1.15±0.7
Docking protein 3	Dok3	1.56±0.7	-1.70±0.4	-1.09±0.06
Dual specificity phosphatase 14	Dusp14	-1.10±0.01	1.43±0.6	1.30±0.03
Eukaryotic translation initiation factor 2B, subunit 1α	Eif2b1	1.05±0.01	-1.11±0.03	-1.06±0.3
**Eukaryotic translation initiation factor 4E binding protein 1**	**Eif4ebp1**	**-2.18±1.3[a]**	**1.09±0.30.6[b]**	**-2.12±1.6[a]**
Excision repair cross-complementing rodent repair deficiency, complementation group 1	Ercc1	-1.32±0.3	-1.13±0.6	-1.50±0.6
Fructose bisphosphatase 1	Fbp1	1.05±0.08[a]	-1.35±1.6	-2.81±1.4
FBJ osteosarcoma oncogene	Fos	-1.26±0.5	-2.20±0.6	-2.77±1.6
Fibroblast growth factor receptor substrate 2	Frs2	-1.62±1.0	1.06±0.6	-1.53±1.3
Fibroblast growth factor receptor substrate 3	Frs3	-1.22±0.6	-1.00±0.6	-1.23±0.06
**Glucose-6-phosphatase, catalytic**	**G6pc**	**2.12±0.01[a]**	**-2.84±2.6[b]**	**-1.36±0.6[c]**
Glucose-6-phosphatase, catalytic, 2	G6pc2	-1.28±0.8	-1.37±1.1	-2.48±1.6
Growth factor receptor bound protein 2-associated protein 1	Gab1	-1.52±0.7	-1.01±0.01	-1.54±0.6
Glucokinase	Gck	-1.44±0.7	-1.14±0.09	-1.65±0.9
Glycerol-3-phosphate dehydrogenase 1 (soluble)	Gpd1	-1.23±0.6	-1.14±0.6	-1.40±0.7
Growth factor receptor bound protein 10	Grb10	-1.97±0.9	1.03±0.5	-1.92±0.6
Growth factor receptor bound protein 2	Grb2	-1.47±0.6	-1.23±0.9	-1.81±1.1
Glycogen synthase kinase 3 beta	Gsk3b	-1.37±0.6	-1.10±0.01	-1.50±0.9
Hexokinase 2	Hk2	1.07±0.06	-1.32±0.6	-1.24±0.04
Harvey rat sarcoma virus oncogene 1	Hras	-1.29±0.7	1.03±0.4	-1.25±0.3
Insulin-like growth factor I receptor	Igf1r	-1.13±0.1	1.07±0.1	-1.05±0.3
Insulin-like growth factor 2	Igf2	-1.14±0.2	-1.14±0.3	-1.30±0.1
Insulin-like growth factor binding protein 1	Igfbp1	-1.28±0.2	-1.06±0.2	-1.05±0.4
Insulin I	Ins1			
Insulin-like 3	Insl3	-1.06±0.5	-1.18±0.2	-1.25±0.9
Insulin receptor substrate 1	Irs1	-1.20±0.2	-1.15±0.1	-1.38±0.2
Insulin receptor substrate 2	Irs2	-1.05±0.1	-1.08±0.3	-1.13±0.2
Jun oncogene	Jun	1.01±0.3	-1.36±0.5	-1.23±0.3
Kruppel-like factor 10	Klf10	1.33±0.7	-2.07±0.4	-1.20±0.5
V-Ki-ras2 Kirsten rat sarcoma viral oncogene homolog	Kras	-1.18±0.4	-1.12±0.3	-1.32±0.3
Low density lipoprotein receptor	Ldlr	-1.15±0.3	-1.10±0.4	-1.27±0.2
Leptin	Lep			
Mitogen-activated protein kinase kinase 1	Map2k1	1.15±0.4	-1.16±0.06	-1.01±0.04
Mitogen-activated protein kinase 1	Mapk1	1.15±0.08	-1.17±0.08	-1.02±0.1
Mechanistic target of rapamycin (serine/threonine kinase)	Mtor	1.04±0.08	-1.25±0.6	-1.20±0.3
Non-catalytic region of tyrosine kinase adaptor protein 1	Nck1	-1.04±0.05	1.07±0.5	1.02±0.3

Nitric oxide synthase 2, inducible	Nos2	1.26±0.3	1.07±0.4	1.35±0.5
Neuropeptide Y	Npy			
Phosphoenolpyruvate carboxykinase 2 (mitochondrial)	Pck2	-1.01±0.3	1.11±0.4	-1.23±0.1
3-phosphoinositide dependent protein kinase 1	Pdpk1	1.09±0.03	-1.17±0.4	-1.07±0.4
Phosphatidylinositol 3-kinase, catalytic, alpha polypeptide	Pik3ca	-1.15±0.4	-1.07±0.4	-1.23±0.5
Phosphatidylinositol 3-kinase, catalytic, beta polypeptide	Pik3cb	-1.40±0.2	-1.10±0.1	-1.53±0.5
Phosphatidylinositol 3-kinase, regulatory subunit, polypeptide 1 (p85 alpha)	Pik3r1	-1.11±0.3	-1.07±0.1	-1.19±0.4
Phosphatidylinositol 3-kinase, regulatory subunit, polypeptide 2 (p85 beta	Pik3r2	-1.07±0.3	-1.08±0.3	-1.15±0.5
Pyruvate kinase liver and red blood cell	Pklr			
Peroxisome proliferator activated receptor gamma	Pparg	-1.14±0.4	-1.55±0.3	-1.76±0.9
Protein phosphatase 1, catalytic subunit, alpha isoform	Ppp1ca	1.17±0.1	-1.07±0.1	1.09±0.1
Protein kinase C, gamma	Prkcg	1.03±0.4	1.24±0.2	1.28±0.1
Protein kinase C, iota	Prkci	-1.10±0.01	-1.00±0.1	-1.10±0.4
Protein kinase C, zeta	Prkcz	-1.79±0.1	1.06±0.5	-1.69±0.4
Prolactin	Prl			
Protein tyrosine phosphatase, non-receptor type 1	Ptpn1	-1.48±0.5	-1.09±0.4	-1.61±0.7
Protein tyrosine phosphatase, receptor type, F	Ptprf	-1.07±0.4	-1.28±0.3	-1.37±0.1
V-raf-leukemia viral oncogene 1	Raf1	-1.12±0.1	-1.09±0.1	-1.23±0.4
Resistin	Retn	-1.13±0.5	-1.19±0.1	-1.46±0.7
Ribosomal protein S6 kinase polypeptide 1	Rps6ka1	-1.05±0.5	-1.14±0.1	-1.20±0.3
Harvey rat sarcoma oncogene, subgroup R	Rras	1.01±0.03	-1.23±0.3	-1.21±0.3
Related RAS viral (r-ras) oncogene homolog 2	Rras2	-1.07±0.05	-1.26±0.4	-1.35±0.3
Serine (or cysteine) peptidase inhibitor, clade E, member 1	Serpine1	1.11±0.8	-1.07±0.9	1.03±0.1
Src homology 2 domain-containing transforming protein C1	Shc1	-1.09±1.0	-1.19±0.7	-1.30±0.7
Solute carrier family 27 (fatty acid transporter), member 4	Slc27a4	1.00±0.2	-1.20±0.7	-1.20±0.8
Solute carrier family 2 (facilitated glucose transporter), member 1	Slc2a1	1.00±0.6	1.03±0.3	1.04±0.02
Sorbin and SH3 domain containing 1	Sorbs1	1.24±0.4	-1.12±0.5	1.11±0.3
Son of sevenless homolog 1 (Drosophila)	Sos1	1.02±0.03	-1.17±0.3	-1.19±0.2
Sterol regulatory element binding transcription factor 1	Srebf1	-1.39±0.04	-1.27±0.3	-1.77±0.7
Thyroglobulin	Tg	-1.28±0.5	1.12±0.04	-1.61±0.05
**Uncoupling protein 1 (mitochondrial, proton carrier)**	**Ucp1**	[a]**1.22**±0.4	[b]**-31.45**±8.4	[b]**-16.7**±4.7
Vascular endothelial growth factor A	Vegfa	-1.33±0.3	-1.55±0.5	-2.06±0.3

[a]Values significantly different (P<0.05) between treatments are indicated by the letters.
